# 1-(4-Benz­yloxy-2-hy­droxy­phen­yl)ethanone

**DOI:** 10.1107/S160053681104637X

**Published:** 2011-11-09

**Authors:** Ya-Tuan Ma, Chong-Lin Yang, Zhen-Shuo Li, Zhong-Qiang Li, Juan-Ning Ding

**Affiliations:** a College of Life Sciences, Northwest A&F University, Yangling Shaanxi 712100, People’s Republic of China; b College of Science, Northwest A&F University, Yangling Shaanxi 712100, People’s Republic of China

## Abstract

The title compound, C_15_H_14_O_3_, has been obtained from the reaction of 2,4-dihy­droxy­acetophenone, potassium carbonate and benzyl bromide. The remaining hy­droxy group is involved in an intra­molecular O—H⋯O hydrogen bond. In the crystal, inter­molecular C—H⋯O contacts occur.

## Related literature

For background to the Williamson reaction in organic synthesis, see: Dermer (1934 ▶). For synthetic procedures for related compounds, see: Mendelson *et al.* (1996 ▶). For a related structure, see: Ma *et al.* (2010 ▶).
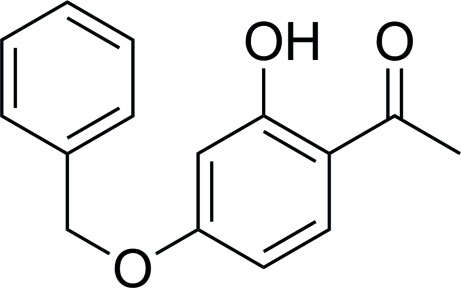


## Experimental

### 

#### Crystal data

C_15_H_14_O_3_
*M* *_r_* = 242.26
Triclinic, 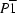

*a* = 5.8433 (7) Å
*b* = 8.0096 (8) Å
*c* = 13.8089 (13) Å
α = 74.061 (1)°
β = 84.589 (1)°
γ = 87.372 (2)°
*V* = 618.54 (11) Å^3^
*Z* = 2
Mo *K*α radiation
μ = 0.09 mm^−1^
*T* = 298 K
0.23 × 0.20 × 0.15 mm

#### Data collection

Siemens SMART CCD area-detector diffractometer
Absorption correction: multi-scan (*SADABS*; Sheldrick, 1996 ▶) *T*_min_ = 0.980, *T*_max_ = 0.987
3169 measured reflections
2167 independent reflections
1291 reflections with *I* > 2σ(*I*)
*R*_int_ = 0.026

#### Refinement

*R*[*F* ^2^ > 2σ(*F* ^2^)] = 0.045
*wR*(*F* ^2^) = 0.114
*S* = 1.02
2167 reflections
165 parameters
H-atom parameters constrained
Δρ_max_ = 0.15 e Å^−3^
Δρ_min_ = −0.14 e Å^−3^

### 

Data collection: *SMART* (Siemens, 1996 ▶); cell refinement: *SAINT* (Siemens, 1996 ▶); data reduction: *SAINT*; program(s) used to solve structure: *SHELXS97* (Sheldrick, 2008 ▶); program(s) used to refine structure: *SHELXL97* (Sheldrick, 2008 ▶); molecular graphics: *SHELXTL* (Sheldrick, 2008 ▶); software used to prepare material for publication: *SHELXTL*.

## Supplementary Material

Crystal structure: contains datablock(s) I, global. DOI: 10.1107/S160053681104637X/im2328sup1.cif

Structure factors: contains datablock(s) I. DOI: 10.1107/S160053681104637X/im2328Isup2.hkl

Supplementary material file. DOI: 10.1107/S160053681104637X/im2328Isup3.cml

Additional supplementary materials:  crystallographic information; 3D view; checkCIF report

## Figures and Tables

**Table 1:** Hydrogen-bond geometry (Å, °)

*D*—H⋯*A*	*D*—H	H⋯*A*	*D*⋯*A*	*D*—H⋯*A*
O2—H2⋯O1	0.82	1.84	2.554 (2)	146
C1—H1*B*⋯O2^i^	0.96	2.52	3.408 (3)	154

Symmetry code: (i) 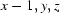.

**Fig. 1.**
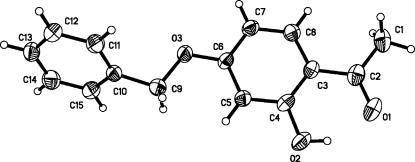
Molecular structure of (I). Displacement ellipsoids are drawn at the 30% probability level.

**Fig. 2.**
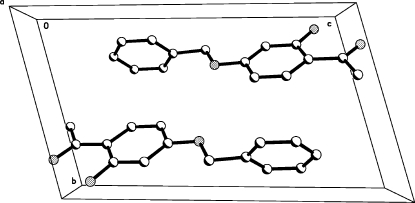
Crystal packing of (I) viewed along the b axis, with hydrogen bonds shown as dashed lines.

## supplementary crystallographic information

### Comment

The Williamson reaction is a very useful transformation in organic synthesis since the products are of value in both industrial and academic applications. It usually involves the employment of an alkali-metal salt of the hydroxy compound and an alkyl halide (Dermer, 1934). Synthetic procedures to ethers derived from 2,4-dihydroxy-acetophenone as well as the structural characterisation of a related molecule have been described before (Mendelson *et al.*, 1996; Ma *et al.*, 2010).

In this paper, we present the title compound, (I), which was synthesized by the reaction of 2, 4-dihydroxy-acetonephenone, potassium carbonate and benzyl bromide. In (I) (Fig. 1), the dihedral angle between the aromatic rings is 53.48 (4)°. The crystal packing exhibits no significantly short intermolecular contacts.

### Experimental

2, 4-Dihydroxy-acetonephenone (4 mmol), potassium carbonate (8 mmol), benzyl bromide (4 mmol), and 40 ml acetone were mixed in a 100 ml flask. After 3 h stirring at 331 K, the crude product was obtained (yield: 78%). Single crystals were obtained by recrystallization from methanol.

### Refinement

The positions of all H atoms were fixed geometrically and refined using a riding model with C—H = 0.93–0.97 Å ,O—H= 0.82 Å, and U_iso_(H) = 1.2–1.5 U_eq_(C, O).

### Crystal data

**Table:**

C_15_H_14_O_3_	*Z* = 2
*M**_r_* = 242.26	*F*(000) = 256
Triclinic, *P*1	*D*_x_ = 1.301 Mg m^−^^3^
Hall symbol: -P 1	Melting point = 378–379 K
*a* = 5.8433 (7) Å	Mo *K*α radiation, λ = 0.71073 Å
*b* = 8.0096 (8) Å	Cell parameters from 945 reflections
*c* = 13.8089 (13) Å	θ = 2.7–25.4°
α = 74.061 (1)°	µ = 0.09 mm^−^^1^
β = 84.589 (1)°	*T* = 298 K
γ = 87.372 (2)°	Triclinic, colourless
*V* = 618.54 (11) Å^3^	0.23 × 0.20 × 0.15 mm

### Data collection

**Table:**

Siemens SMART CCD area-detector diffractometer	2167 independent reflections
Radiation source: fine-focus sealed tube	1291 reflections with *I* > 2σ(*I*)
graphite	*R*_int_ = 0.026
phi and ω scans	θ_max_ = 25.0°, θ_min_ = 2.7°
Absorption correction: multi-scan (*SADABS*; Sheldrick, 1996)	*h* = −6→6
*T*_min_ = 0.980, *T*_max_ = 0.987	*k* = −9→9
3169 measured reflections	*l* = −16→15

### Refinement

**Table:**

Refinement on *F*^2^	Primary atom site location: structure-invariant direct methods
Least-squares matrix: full	Secondary atom site location: difference Fourier map
*R*[*F*^2^ > 2σ(*F*^2^)] = 0.045	Hydrogen site location: inferred from neighbouring sites
*wR*(*F*^2^) = 0.114	H-atom parameters constrained
*S* = 1.02	*w* = 1/[σ^2^(*F*_o_^2^) + (0.0468*P*)^2^] where *P* = (*F*_o_^2^ + 2*F*_c_^2^)/3
2167 reflections	(Δ/σ)_max_ < 0.001
165 parameters	Δρ_max_ = 0.15 e Å^−^^3^
0 restraints	Δρ_min_ = −0.14 e Å^−^^3^

### Special details

**Table:**

Geometry. All e.s.d.'s (except the e.s.d. in the dihedral angle between two l.s. planes) are estimated using the full covariance matrix. The cell e.s.d.'s are taken into account individually in the estimation of e.s.d.'s in distances, angles and torsion angles; correlations between e.s.d.'s in cell parameters are only used when they are defined by crystal symmetry. An approximate (isotropic) treatment of cell e.s.d.'s is used for estimating e.s.d.'s involving l.s. planes.
Refinement. Refinement of *F*^2^ against ALL reflections. The weighted *R*-factor *wR* and goodness of fit *S* are based on *F*^2^, conventional *R*-factors *R* are based on *F*, with *F* set to zero for negative *F*^2^. The threshold expression of *F*^2^ > σ(*F*^2^) is used only for calculating *R*-factors(gt) *etc*. and is not relevant to the choice of reflections for refinement. *R*-factors based on *F*^2^ are statistically about twice as large as those based on *F*, and *R*- factors based on ALL data will be even larger.

### Fractional atomic coordinates and isotropic or equivalent isotropic displacement parameters (Å^2^)

**Table:**

	*x*	*y*	*z*	*U*_iso_*/*U*_eq_	
O1	0.6013 (3)	0.8182 (2)	−0.02477 (10)	0.0812 (5)	
O2	0.8544 (3)	0.91909 (18)	0.08846 (10)	0.0723 (5)	
H2	0.8070	0.9162	0.0349	0.108*	
O3	0.6839 (2)	0.71044 (16)	0.44284 (9)	0.0584 (4)	
C1	0.2766 (4)	0.6428 (3)	0.03392 (16)	0.0804 (7)	
H1A	0.2655	0.6613	−0.0372	0.121*	
H1B	0.1382	0.6848	0.0635	0.121*	
H1C	0.2971	0.5210	0.0653	0.121*	
C2	0.4770 (4)	0.7384 (3)	0.04958 (16)	0.0627 (6)	
C3	0.5296 (3)	0.7344 (2)	0.15150 (13)	0.0499 (5)	
C4	0.7175 (3)	0.8243 (2)	0.16617 (13)	0.0529 (5)	
C5	0.7750 (3)	0.8181 (2)	0.26222 (13)	0.0520 (5)	
H5	0.9021	0.8771	0.2705	0.062*	
C6	0.6417 (3)	0.7236 (2)	0.34562 (13)	0.0479 (5)	
C7	0.4525 (3)	0.6338 (2)	0.33393 (14)	0.0544 (5)	
H7	0.3636	0.5700	0.3902	0.065*	
C8	0.3993 (3)	0.6409 (2)	0.23848 (14)	0.0566 (5)	
H8	0.2719	0.5815	0.2310	0.068*	
C9	0.8747 (3)	0.8038 (3)	0.45748 (14)	0.0605 (6)	
H9A	0.8578	0.9257	0.4220	0.073*	
H9B	1.0162	0.7594	0.4301	0.073*	
C10	0.8863 (3)	0.7845 (2)	0.56755 (14)	0.0501 (5)	
C11	0.7172 (3)	0.8552 (3)	0.62181 (15)	0.0618 (6)	
H11	0.5887	0.9088	0.5905	0.074*	
C12	0.7353 (4)	0.8478 (3)	0.72185 (15)	0.0655 (6)	
H12	0.6197	0.8961	0.7574	0.079*	
C13	0.9239 (4)	0.7690 (3)	0.76880 (16)	0.0654 (6)	
H13	0.9373	0.7646	0.8360	0.078*	
C14	1.0919 (4)	0.6970 (3)	0.71643 (17)	0.0688 (6)	
H14	1.2196	0.6430	0.7482	0.083*	
C15	1.0730 (4)	0.7039 (3)	0.61631 (15)	0.0606 (6)	
H15	1.1878	0.6535	0.5815	0.073*	

### Atomic displacement parameters (Å^2^)

**Table:**

	*U*^11^	*U*^22^	*U*^33^	*U*^12^	*U*^13^	*U*^23^
O1	0.1067 (13)	0.0896 (12)	0.0446 (9)	0.0045 (10)	−0.0021 (9)	−0.0160 (8)
O2	0.0836 (10)	0.0796 (11)	0.0449 (8)	−0.0142 (8)	0.0097 (7)	−0.0051 (7)
O3	0.0708 (9)	0.0625 (9)	0.0418 (8)	−0.0226 (7)	−0.0022 (6)	−0.0115 (6)
C1	0.0932 (18)	0.0912 (18)	0.0669 (15)	0.0076 (15)	−0.0257 (13)	−0.0336 (13)
C2	0.0768 (16)	0.0593 (14)	0.0544 (14)	0.0154 (12)	−0.0087 (12)	−0.0211 (11)
C3	0.0601 (13)	0.0478 (12)	0.0422 (12)	0.0036 (10)	−0.0029 (9)	−0.0139 (9)
C4	0.0627 (14)	0.0488 (12)	0.0410 (12)	0.0001 (10)	0.0065 (10)	−0.0057 (9)
C5	0.0569 (13)	0.0515 (12)	0.0464 (12)	−0.0099 (10)	−0.0014 (10)	−0.0106 (9)
C6	0.0593 (12)	0.0441 (11)	0.0385 (11)	−0.0048 (10)	0.0007 (9)	−0.0096 (9)
C7	0.0612 (13)	0.0548 (13)	0.0461 (12)	−0.0141 (10)	0.0056 (10)	−0.0134 (9)
C8	0.0581 (13)	0.0589 (13)	0.0558 (13)	−0.0075 (10)	−0.0035 (10)	−0.0202 (10)
C9	0.0640 (14)	0.0650 (14)	0.0533 (13)	−0.0178 (11)	−0.0017 (10)	−0.0158 (10)
C10	0.0537 (12)	0.0511 (12)	0.0466 (12)	−0.0088 (10)	−0.0041 (10)	−0.0137 (9)
C11	0.0546 (13)	0.0719 (15)	0.0578 (14)	0.0027 (11)	−0.0098 (10)	−0.0147 (11)
C12	0.0641 (14)	0.0770 (16)	0.0577 (14)	−0.0032 (12)	0.0022 (11)	−0.0245 (11)
C13	0.0768 (16)	0.0720 (16)	0.0485 (13)	−0.0132 (13)	−0.0077 (12)	−0.0158 (11)
C14	0.0660 (15)	0.0761 (16)	0.0606 (15)	0.0005 (12)	−0.0168 (12)	−0.0088 (12)
C15	0.0606 (14)	0.0612 (14)	0.0595 (14)	0.0012 (11)	0.0002 (11)	−0.0178 (10)

### Geometric parameters (Å, °)

**Table:**

O1—C2	1.238 (2)	C7—H7	0.9300
O2—C4	1.347 (2)	C8—H8	0.9300
O2—H2	0.8200	C9—C10	1.493 (2)
O3—C6	1.362 (2)	C9—H9A	0.9700
O3—C9	1.430 (2)	C9—H9B	0.9700
C1—C2	1.491 (3)	C10—C15	1.377 (3)
C1—H1A	0.9600	C10—C11	1.379 (3)
C1—H1B	0.9600	C11—C12	1.380 (2)
C1—H1C	0.9600	C11—H11	0.9300
C2—C3	1.460 (3)	C12—C13	1.371 (3)
C3—C4	1.400 (3)	C12—H12	0.9300
C3—C8	1.404 (2)	C13—C14	1.365 (3)
C4—C5	1.386 (2)	C13—H13	0.9300
C5—C6	1.383 (2)	C14—C15	1.383 (3)
C5—H5	0.9300	C14—H14	0.9300
C6—C7	1.392 (2)	C15—H15	0.9300
C7—C8	1.368 (2)		
			
C4—O2—H2	109.5	C7—C8—H8	118.8
C6—O3—C9	117.04 (14)	C3—C8—H8	118.8
C2—C1—H1A	109.5	O3—C9—C10	109.89 (15)
C2—C1—H1B	109.5	O3—C9—H9A	109.7
H1A—C1—H1B	109.5	C10—C9—H9A	109.7
C2—C1—H1C	109.5	O3—C9—H9B	109.7
H1A—C1—H1C	109.5	C10—C9—H9B	109.7
H1B—C1—H1C	109.5	H9A—C9—H9B	108.2
O1—C2—C3	120.3 (2)	C15—C10—C11	118.14 (18)
O1—C2—C1	119.2 (2)	C15—C10—C9	120.72 (17)
C3—C2—C1	120.5 (2)	C11—C10—C9	121.03 (18)
C4—C3—C8	116.92 (17)	C10—C11—C12	121.11 (19)
C4—C3—C2	120.48 (19)	C10—C11—H11	119.4
C8—C3—C2	122.6 (2)	C12—C11—H11	119.4
O2—C4—C5	116.22 (19)	C13—C12—C11	119.94 (19)
O2—C4—C3	122.26 (17)	C13—C12—H12	120.0
C5—C4—C3	121.51 (17)	C11—C12—H12	120.0
C6—C5—C4	119.41 (19)	C14—C13—C12	119.7 (2)
C6—C5—H5	120.3	C14—C13—H13	120.2
C4—C5—H5	120.3	C12—C13—H13	120.2
O3—C6—C5	123.69 (18)	C13—C14—C15	120.3 (2)
O3—C6—C7	115.64 (16)	C13—C14—H14	119.8
C5—C6—C7	120.67 (17)	C15—C14—H14	119.8
C8—C7—C6	119.02 (18)	C10—C15—C14	120.81 (19)
C8—C7—H7	120.5	C10—C15—H15	119.6
C6—C7—H7	120.5	C14—C15—H15	119.6
C7—C8—C3	122.5 (2)		
			
O1—C2—C3—C4	1.6 (3)	C5—C6—C7—C8	−0.3 (3)
C1—C2—C3—C4	−179.90 (17)	C6—C7—C8—C3	0.5 (3)
O1—C2—C3—C8	−177.70 (18)	C4—C3—C8—C7	−1.0 (3)
C1—C2—C3—C8	0.8 (3)	C2—C3—C8—C7	178.29 (16)
C8—C3—C4—O2	−179.70 (16)	C6—O3—C9—C10	176.25 (14)
C2—C3—C4—O2	1.0 (3)	O3—C9—C10—C15	117.1 (2)
C8—C3—C4—C5	1.3 (3)	O3—C9—C10—C11	−66.5 (2)
C2—C3—C4—C5	−178.00 (16)	C15—C10—C11—C12	0.9 (3)
O2—C4—C5—C6	179.84 (16)	C9—C10—C11—C12	−175.56 (18)
C3—C4—C5—C6	−1.1 (3)	C10—C11—C12—C13	0.0 (3)
C9—O3—C6—C5	1.4 (2)	C11—C12—C13—C14	−0.6 (3)
C9—O3—C6—C7	−178.88 (15)	C12—C13—C14—C15	0.3 (3)
C4—C5—C6—O3	−179.73 (15)	C11—C10—C15—C14	−1.2 (3)
C4—C5—C6—C7	0.6 (3)	C9—C10—C15—C14	175.30 (19)
O3—C6—C7—C8	−179.99 (15)	C13—C14—C15—C10	0.6 (3)

### Hydrogen-bond geometry (Å, °)

**Table:**

*D*—H···*A*	*D*—H	H···*A*	*D*···*A*	*D*—H···*A*
O2—H2···O1	0.82	1.84	2.554 (2)	146
C1—H1B···O2^i^	0.96	2.52	3.408 (3)	154

Symmetry codes: (i) *x*−1, *y*, *z*.
